# Dynamics of h-Shaped Pulse to GHz Harmonic State in a Mode-Locked Fiber Laser

**DOI:** 10.3390/mi16121358

**Published:** 2025-11-29

**Authors:** Lin Wang, Guoqing Hu, Yan Wang, Guangwei Chen, Liang Xuan, Zhehai Zhou, Jun Yu

**Affiliations:** 1 East China Engineering Science & Technology Co., Ltd., Hefei 535019, China; wanglin@chinaecec.com (L.W.); wangyan@chinaecec.com (Y.W.); xuanliang@chinaecec.com (L.X.); yujun@chinaecec.com (J.Y.); 2 Key Laboratory of Modern Optoelectronic Measurement Technology in Mechanical Industry, Beijing Information Science and Technology University, Beijing 100192, China; guoqing2011@foxmail.com (G.H.); zhouzhehai@bistu.edu.cn (Z.Z.)

**Keywords:** mode-locked fiber laser, GHz, Ginzburg-Landau equation, bunch solitons

## Abstract

We experimentally and through simulations demonstrate a passively mode-locked fiber laser based on nonlinear polarization rotation, which generates the evolution from h-shaped pulses to GHz harmonic trains. When the polarization angle is continuously changed, the h-shaped pulse sequentially evolves into multiple pulses, bunched solitons, and harmonic pulses. The maximum order of harmonic trains obtained in experiments is 120, corresponding to the repetition frequency of 1.03996 GHz. The coupled Ginzburg-Landau equation and two-time-scale approach to gain is provided to characterize the laser physics. The fast and slow evolution of gain contributes to the stabilization and length of one soliton pattern, respectively. The proposed fiber laser is cost effective and easy to implement, providing a potential way to study soliton dynamics in depth.

## 1. Introduction

Optical solitons in lasers, as special solutions of localized nonlinear waves, are treated as ideal core elements in many physical settings, such as condensed matter, telecommunication systems, and optical information storage [1,2,3]. Passively mode-locked fiber lasers are ideal candidates for generating optical solitons. Precise control of dynamical soliton behaviors allows manipulation of fiber laser fields and the production of self-stabled light structures, for example, phase-locked bound solitons, synchronized multi-wavelength solitons, and soliton crystals [4,5,6]. These interactions can be realized by carefully engineering the cavity parameters or implementing intracavity modulation, which is experimentally achieved by modulating birefringence, nonlinear effects, nonlinear saturable absorption, and dispersion distribution. Soliton spacing determines the strength of the interactions. Long-range soliton interactions can bind solitons, making the separations tens or even thousands of picoseconds. K. Sulimany et al. demonstrate that long-range Casimir-like interactions arise from the nonlinear overlap between pulses and a random quasi-cw background and play a major role in controlling dynamics of soliton rain and bunch collisions [7]. The binding force between acoustic waves and solitons can trap pulses with ns-level separations, corresponding to acoustic modes in fiber, and generates cavity solitons or countable bunched solitons [8,9]. For multiple optical solitons with short-range interaction, the binding of interacting solitons arises from the pulse tail field, generating bound soliton, soliton bunches or periodic soliton patterns. G. Herink et al. experimentally investigated phase-locked soliton molecules using a real-time time-stretch technique, tracking the fast internal motion of bound states with different oscillating separation and phase [10]. A. Andrianov et al. presented a stable periodic soliton train, i.e., soliton crystals, using a highly asymmetric Mach-Zehnder interferometer in a passive fiber resonator [11]. In high energy regimes, single soliton will split into multiple pulses due to the soliton area theory and energy quantization effect, forming bunched solitons once they are in phase. Generally, the pulses in bunched solitons are randomly spaced in the cavity [12]. In order to arrange the pulses, various techniques have been implemented, such as additive phase/amplitude modulation, acousto-optic modulation, and gain depletion, allowing the establishment of harmonic mode-locked pulses [13,14].

In addition to these discrete and distinguishable multiple pulse patterns, large duration condensed-phase pulses denoted as soliton rains, dissipative soliton resonance (DSR), etc., have gained widespread attention. By controlling the polarization states in the cavity, isolated solitons can be captured or released by condensed-phase pulses, which can have even tens of nanoseconds of temporal duration [15]. DSR pulses own a rectangular temporal profile, and exhibit wave-breaking-free features with increasing pump power [16]. Recently, an intriguing pulse pattern named the “h-shaped” pulse has been the focus of attention, featuring a prominent leading edge and a lower-amplitude trailing portion [16,17]. The prominent leading edge results from the high nonlinearity and partial clamping effect [16]. The temporal envelope of h-shaped pulses exhibits similar evolutionary characteristics to DSR, when the pump increases. Theoretical approaches have been implemented to predict multiple soliton formation in both negative and positive dispersion regimes, including models based on the scalar and coupled Ginzburg–Landau equations [1,18]. However, these theoretical predictions are predicated on slow gain relaxation times, limiting them to only MHz-level repetition frequency and only a few-pulse patterns [19]. The nature of the h-shaped pulse is not yet clear.

In this work, we experimentally and through simulations investigate the generation of h-shaped pulses and their evolution from h-shaped pulses to multiple pulses, eventually reaching harmonic states. The stable h-shaped pulse has an 8.66 MHz repetition frequency. The pulse maintains an almost constant spectral width and center wavelength, but its temporal width gradually increases up to ~55 ns as the pump current is increased to 450 mA. Because of the depletion and recovery of gain, the h-shaped pulse will evolve into a multi-pulse state, i.e., soliton bunches with altered polarization states. With suitable polarization states, the harmonic pulse can be obtained, and the maximum harmonic repetition frequency is ~1.04 GHz. The simulation is also implemented to study in depth the phase-locking mechanism of multiple pulses.

## 2. Methods

**Figure 1:**
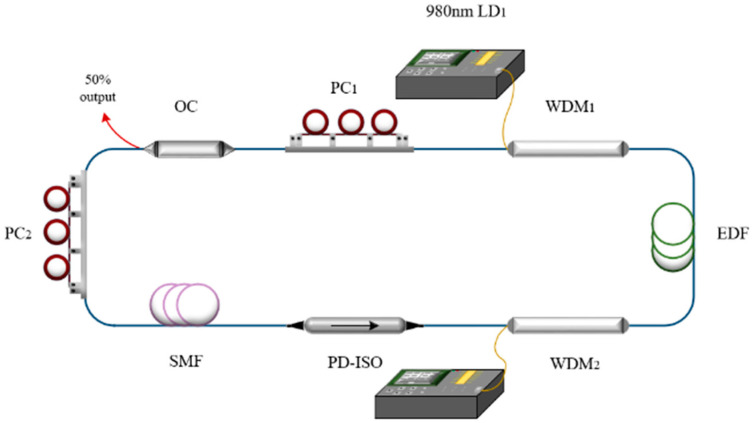
Schematic setup of mode-locked fiber laser. LD, laser diode; WDM, wavelength division multiplexer; EDF, Er-doped fiber; OC, optical coupler; PD-ISO, polarization dependent isolator; PC, polarization controller; SMF, single mode fiber.

The experimental setup of the Er-doped mode-locked fiber laser is shown in Figure 1. The nonlinear polarization rotation (NPR) effect is used to achieve passive mode-locking. The total cavity length of the ring laser is ~23 m, with a ~10 m erbium-doped fiber (EDF) used as the gain medium. In order to adjust the intracavity dispersion, we added an additional 2 m single-mode fiber (SMF) into the cavity. The group velocity dispersion coefficient of EDF is −17.8 ps (nm·km)^−1^ while the SMF and HI1060 tail fibers of the device are 17 ps (nm·km)^−1^ and 5.6 ps (nm·km)^−1^. The total dispersion β2 in the cavity is −0.009 ps2, which indicates the laser operating in a near-zero dispersion region. In order to fully pump EDF, the laser adopts a dual-pump structure via two wavelength division multiplexers (WDM). The 50% output is extracted from the cavity for measurement. Two polarization controllers (PC) and a polarization dependent isolator (PD-ISO) are added into the cavity to achieve the NPR effect. The output spectra and radio frequency (RF) are measured with a spectrum analyzer (Anritsu, MS9710C, made in Kanagawa Prefecture, Japan) and a frequency spectrograph (KEYSIGHT, N9320B, made in Santa Rosa, CA, USA), respectively. In addition, the output pulse temporal sequence is measured by a 4-GHz, 20-GS/s oscilloscope (KEYSIGHT, DSO99404A, made in Santa Rosa, CA, USA) and an Autocorrelator (APE, PulseCheck SM2000, made in Berlin, Germany).

In our experiments, the polarization states are characterized are inferred from three-paddle PC adjustments, with the loop configuration being 2-4-2 turns. The three-paddle PC consists of a quarter-wave plate, a half-wave plate, and another quarter-wave plate connected in series, enabling the conversion of any polarization state into another. The phase delay of each paddle can be described as

(1) $$\phi =\frac{2\pi^{2}aNd^{2}}{\lambda D}$$

where *N*, *d* = 125 μm, and *D* = 35 mm are the number of winding turns, the fiber cladding diameter, and the winding diameter. *a* = 0.133 for silica fiber.

## 3. Results

### 3.1. Experimental Results

**Figure 2:**
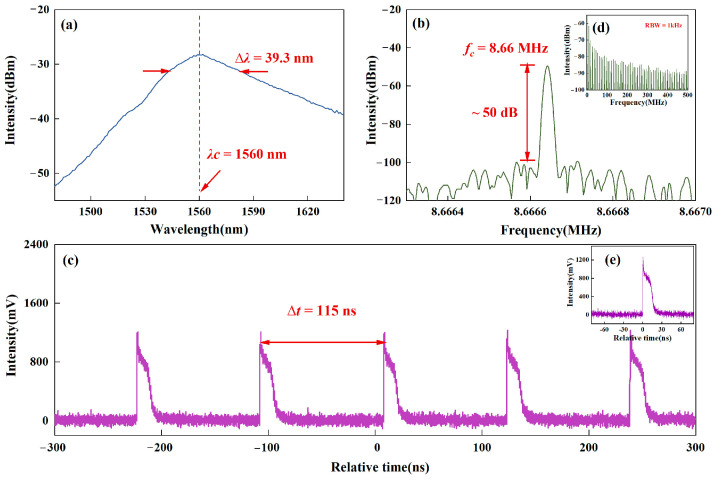
The h-shaped pulse output characteristics. (**a**) The output spectrum information, and (**b**,**d**) RF with the RBW of 10 Hz, and 500 MHz, respectively, (**c**,**e**) temporal information with time ranges of 600 ns and 160 ns, respectively.

Increasing the pump current to Pump 1 = 300 mA and Pump 2 = 150 mA, a special mode-locked pulse is obtained. The output characteristics of the typical h-shaped pulse are shown in Figure 2. The output spectrum displays a wide and relatively smooth envelope, as shown in Figure 2a. The central wavelength is 1560 nm, with a 3 dB bandwidth of 39.3 nm. Figure 2b and Figure 2d display the RF traces with span/RBW of 1 kHz/10 Hz, 500 MHz/1 kHz, respectively. The fundamental frequency of the output pulses is measured as about 8.66 MHz and the signal-to-noise ratio (SNR) is about 50 dB. Figure 2d shows a period-like, gradually decaying frequency modulation, which is weaker than DSR because of the prominent leading edge of the pulse. Figure 2c records the time-domain train of the h-shaped pulse, with a time interval Δt of 115 ns between the two h-shaped pulses, corresponding to the length of the cavity. The waveform of a single pulse is shown in Figure 2e, resembling the lowercase letter “h”, with a prominent leading edge and a lower trailing portion.

**Figure 3:**
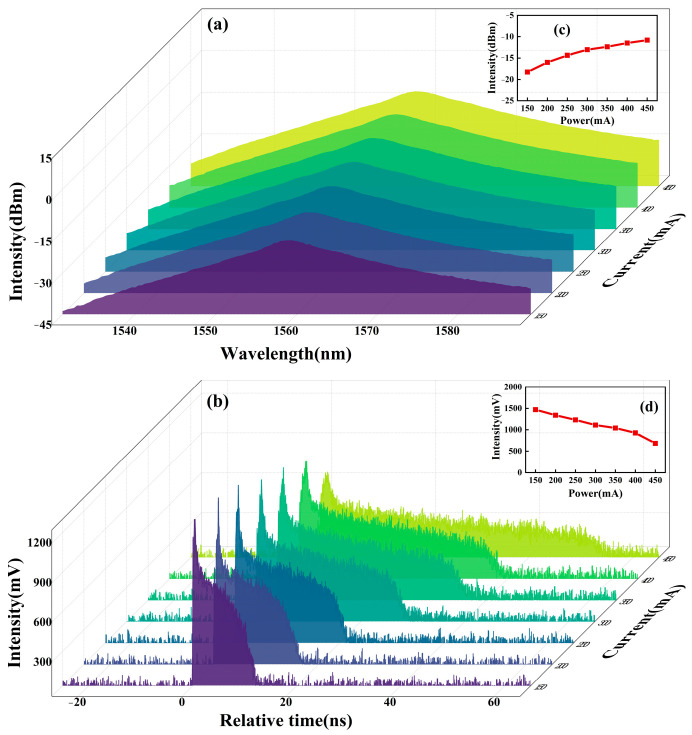
Evolution of h-shaped pulse under different pump power. (**a**) Output spectrum, (**b**) single temporal envelope, (**c**) spectral intensity and (**d**) pulse intensity variation curves with pump.

With Pump 1 fixed at 200 mA and Pump 2 varied from 150 mA to 400 mA, the evolution of the h-shaped pulse envelope and optical spectrum is recorded in Figure 3. Figure 3a shows the corresponding spectral evolution under different pump currents. As Pump 2 is increased, the 3 dB bandwidth and the center wavelength remain almost unchanged, at approximately ~8 nm and ~1560 nm, respectively. However, the spectral intensity changes significantly, as shown in Figure 3c, showing an approximately linear upward trend. The corresponding pulse envelope evolution is shown in Figure 3b. Time width of the pulse trailing continuously increases as the pump current increases, accompanied by a nearly linear decrease in the overall amplitude, as shown in Figure 3d.

**Figure 4:**
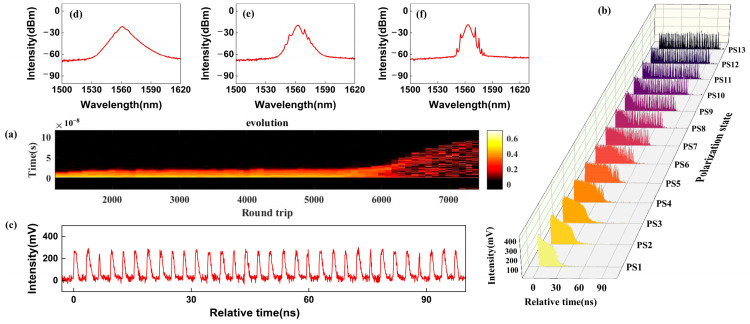
The polarization-related characterization of the h-type pulse. (**a**) The evolution process, (**b**) time-domain evolution, (**c**) a multi-pulse sequence, (**d**–**f**) and the output spectra for different PS.

By fixing Pump 1 and Pump 2, the polarization-dependent characterizations are studied by adjusting the polarization angels in the cavity. We observed that the h-shaped pulse can be transformed into multiple pulses with suitable polarization states as shown in Figure 4. In Figure 4a, the evolution from h-shaped pulses to multi-pulses was recorded through the rapid storage of high-speed oscilloscopes. It can be seen that the pulses begin to split at ~5600 cycles. With fine tuning the polarization states (PS), different temporal envelopes are obtained, as shown in Figure 4b. As the PC is tuned from PS1 to PS3, the duration of the trailing edge of the pulse increases, while the leading edge of the pulse gradually disappears and the overall amplitude decreases. The h-shaped pulse gradually evolves into a form similar to DSR. Continuing to adjust the PC to PS4, the small spikes begin to appear at the trailing edge of the pulse. From PS3 to PS13, the number of small spikes at the trailing edge of the pulse gradually increases and begins to split from the main pulse to form an independent small pulse. By continuously changing the polarization state, it eventually evolves into a completely split state. The stable multiple pulse train is shown in Figure 4c. When the pulse begins to split, it is not approximately equidistant in Figure 4c, but rather irregular. It should be noted that the pulse evolution process is achieved by rotating one of the six polarizers in the two PCs, while keeping the other five polarizers fixed.

Figure 4d–f shows the captured spectra during the evolution process. Figure 4d records the initial part of the evolution which corresponds to the PS4 in Figure 4b. The spectrum in this state is no longer as smooth as in Figure 2a, and there are signs of sidebands on both sides of the central wavelength. When the polarizer is adjusted to a partially split state of pulse, the corresponding spectral information is shown in Figure 4e, and the 3 dB bandwidth decreases with obvious sidebands. When the pulse is completely split, the spectrum transforms into a mode-locking state similar to that of a cavity with negative dispersion as shown in Figure 4f. During the whole evolution process, the 3 dB bandwidth of the spectrum is gradually narrowed.

**Figure 5:**
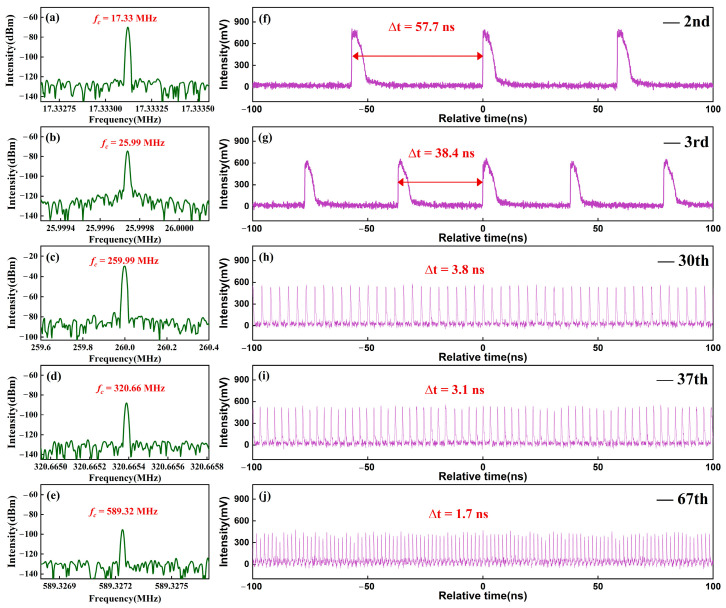
Polarization-induced harmonic mode-locked pulse trains. (**a**–**e**) RF spectra, and (**f**–**j**) pulse trains in 2, 3, 30, 37, and 67-order harmonic states.

**Figure 6:**
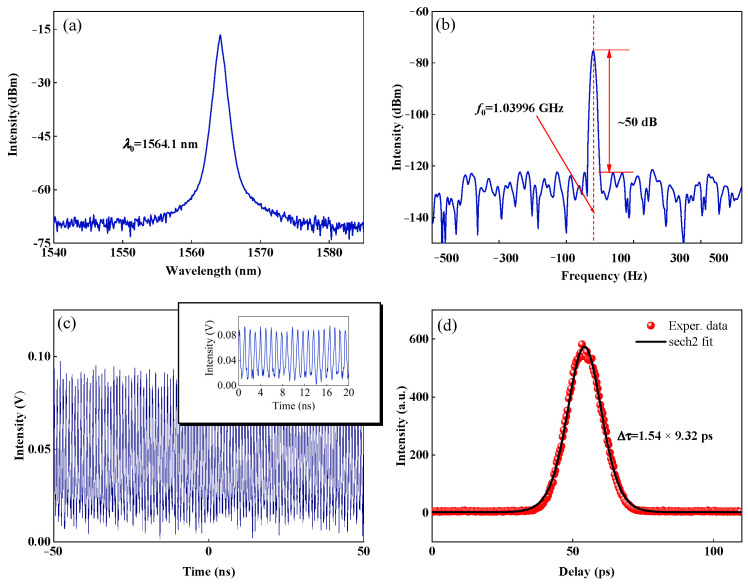
GHz pulse output characteristics. (**a**) Output spectrum, (**b**) RF spectrum, (**c**) pulse trains, and (**d**) autocorrelation trace.

With suitable polarization states, diverse order harmonic mode-locked states are obtained, as shown in Figure 5. Different from changing only one polarizer in Figure 4, the realization of different harmonic mode-locking states is the result of changing several polarizers at the same time. Figure 5f–j shows the pulse trains of the 2nd, 3rd, 30th, 37th, and 67th harmonic mode-locking states, corresponding to the frequency components of 17.33 MHz, 25.99 MHz, 259.99 MHz, 320.66 MHz, and 589.32 MHz in Figure 5a–e, respectively. As the harmonic order increases, the temporal span of a single pulse envelope gradually decreases. When the pump current is increased to 560 mA, higher-order mode-locked pulses with a repetition frequency up to the GHz-level are generated, as show in Figure 6. The center wavelength of the GHz pulse is 1564.1 nm, with a 3 dB spectral bandwidth of 0.6 nm. The RF spectrum is measured with a 10 Hz resolution and a 1 kHz span, with an RF of 1.03996 GHz and an SNR of ~50 dB. The temporal distribution of output intensity on a 100 ns scale is presented in Figure 6c. From an enlargement of Figure 6c, we can see that the pulse distribution is uniform. The autocorrelation trace is shown in Figure 6d. The pulse is fitted with a sech-pulse shaped profile, yielding a pulse duration of Δτ = 9.32 ps. The corresponding chirp parameter is calculated as ~0.29. Thus, the time-bandwidth product is about 0.69, about 2.2 times higher than the Fourier transform limit. To test the stability of the harmonic pulses, we conducted continuous monitoring of the output pulses for 9 h. After 9 h, the harmonic pulse transformed into a multi-pulse state similar to that shown in Figure 4c.

### 3.2. Simulation and Discussion

To deeply understand of the mechanisms of polarization-dependent transition and production of harmonic pulses, the coupled Ginzburg–Landau equations and two-time scale approach to gain are used to describe nonlinear propagation of the dynamics of multiple solitons [1,20].

(2) $$\left\{\begin{array}{c} \frac{\partial u_{x}}{\partial z}=-i\frac{\Delta \beta }{2}u_{x}+\delta \frac{\partial u_{x}}{\partial t}-i\frac{\beta_{2}}{2}\frac{\partial^{2}u_{x}}{\partial t^{2}}+i\gamma \left({\left|u_{x}\right|}^{2}+\frac{2}{3}{\left|u_{y}\right|}^{2}\right)u_{x}+\frac{g}{2}u_{x}+\frac{g}{2\Omega_{g}^{2}}\frac{\partial^{2}u_{x}}{\partial t^{2}}\\ \frac{\partial u_{y}}{\partial z}=i\frac{\Delta \beta }{2}u_{y}-\delta \frac{\partial u_{y}}{\partial t}-i\frac{\beta_{2}}{2}\frac{\partial^{2}u_{y}}{\partial t^{2}}+i\gamma \left({\left|u_{y}\right|}^{2}+\frac{2}{3}{\left|u_{x}\right|}^{2}\right)u_{y}+\frac{g}{2}u_{y}+\frac{g}{2\Omega_{g}^{2}}\frac{\partial^{2}u_{y}}{\partial t^{2}}\\ \frac{\partial g}{\partial t}=-\frac{g}{\tau_{g}}-\frac{gP}{E_{g}}+\Lambda \end{array}\right.$$

where *u*_x_ and *u*_y_ represent the amplitude envelope of the two vertical components. *β*_2_, *γ*, *g,* and Ω_g_ are the group velocity dispersion, nonlinear parameter, gain, and gain bandwidth, respectively. Δ*β = 2π*Δ*n/λ*, 2*δ* = Δ*β/2πc* denotes the wave number difference and inverse group velocity difference between the two components, respectively. Δ*n* denotes the difference in refractive index. *P*, *E*_g_, *τ*_g_, and Λ are instantaneous the pulse intensity, gain saturable energy, relaxation time, and pump coefficient, respectively. Considering the small ratio *η* of pulse duration to *τ*_g_, the rate equation of *g* contains two different terms that vary rapidly and slowly over time. Here we introduce a fast time variable *T* and a slow one *τ* to describe the pulse envelope and gain relaxation, respectively. Thus, the gain *g* can be expressed as $g=g_{\tau }+\eta g_{T}$, where *g*_τ_ and *g*_T_ denote slow- and fast-varying parts of gain, respectively. The laser gain can be expressed as [12,20]

(3) $$\left\{\begin{array}{c} g_{\tau }=\int_{\tau_{0}}^{\tau_{0}+T_{r}}\left(-\frac{g_{\tau }}{\tau_{g}}-\frac{g_{\tau }<P>}{E_{g}}+\Lambda \right)d\tau^{\prime }\\ g_{T}=-\frac{g_{\tau }}{\tau_{g}}\int_{-\infty }^{T}\left(P-<P>\right)dT^{\prime } \end{array}\right.$$

where *T*_r_ is the pulse roundtrip time, and <P> denotes the average intensity over a period of pulse pattern. The length of pulse pattern is determined by *g*_τ_. The depletion of net gain by each pulse in pattern is 2*δG*, which is related to the parameters of peak power *P*_p_, pulse duration, cavity length, and slow-varying gain [12,19]. In the time interval Δ*τ* between two pulse patterns, the gain will gradually recover to the initial value due to a pumping and relaxation effect. The variation of gain in relaxation time is about Δ*g* = Λ·Δ*τ* = *N*·2*δG* = *N*·[exp(2*g*_τ_L)−1]*ξPτ*, where *L*, *ξ*, *P*, and *τ* are the cavity length, saturable parameter, pulse power, and pulse duration, respectively. Thus, the number of pulses in one pattern is *N* = Δ*g*/2*δG*. When the pump coefficient Λ is 7.7 × 10^3^ (m·s)^−1^, and the time interval is about 570 ps, the gain recovered in the absence of pulse Δg~4.4 × 10^−6^ m^−1^. When the pulse pattern consisting of 28 single pulses with a duration of 1 ps, the gain depletion consecutive to a single soliton is 2δG~1.5 × 10^−7^ m^−1^. Here, we present several sets of depletion gain values under different pump and rotation angles in simulation, as shown in Table 1.

**Figure 7:**
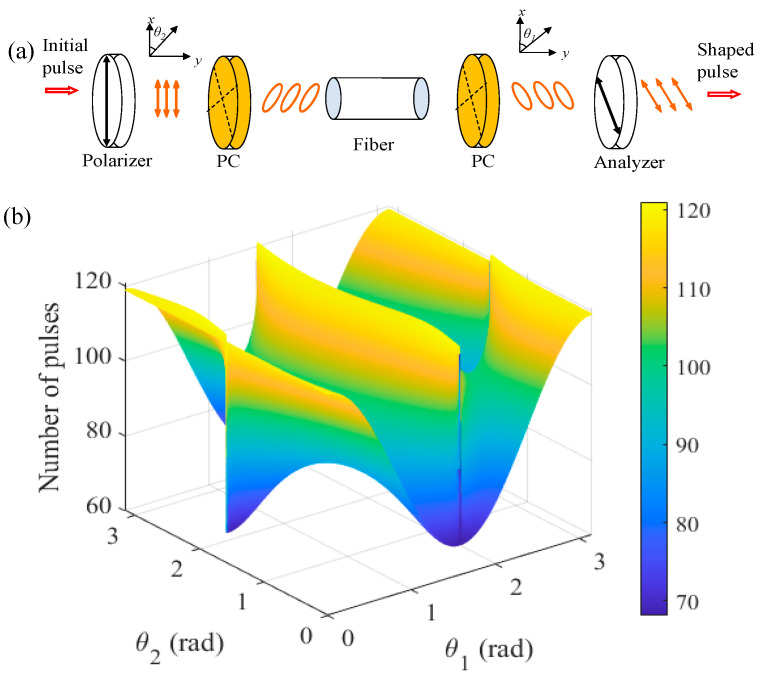
The number of pulses in the pulse pattern tuning characteristic. (**a**) Orientation angles *θ*_1_, *θ*_2_ in the NPR structure, and (**b**) simulation results of the pulse number as a function of the angles *θ*_1_, *θ*_2_.

The laser used in the simulation is a simplified model based on the cavity structure shown in Figure 1. The NPR model is plotted in Figure 7a, which is widely applied in NPR-based laser systems. The rate equation employed to describe the gain dynamics of gain media is applicable to any laser system based on doped optical fibers. Considering the experimental results, the NPR structure is modeled in Figure 7a to investigate the role of orientation angles on the pulse pattern. *θ*_1_ and *θ*_2_ are the angles between the passing axes of the polarizer/analyzer and the fiber eigenaxis. When (*θ*_1_, *θ*_2_) is varied from (0, 0) to (π, π), the number of pulses in a pattern can be tuned, as show in Figure 7b. In the simulation, the following parameters are selected to match experimental conditions: *λ*_0_ = 1560 nm, Ω_g_ = 30 nm, *g*_0_ = 6 dB, and Δ*n* = 1.6 × 10^−6^. For the single-mode fiber, *L* = 13.1 m, *γ* = 0.0013 (W·m)^−1^, and *β*_2_ = −0.0219 ps^2^/m. For EDF, *L* = 10.0 m, *γ* = 0.0013 (W·m)^−1^, and *β*_2_ = 0.0228 ps^2^/m. When Λ is set as 1.62 × 10^25^ (m^3^·s)^−1^, the number of pulses in the pulse pattern tuning characteristic is shown in Figure 7b. We can note that the maximum number of pulses in one pulse pattern is 120, for example, with the (*θ*_1_, *θ*_2_) of (3.10, 1.47), which corresponds to the 1.034 GHz state shown in Figure 6.

**Figure 8:**
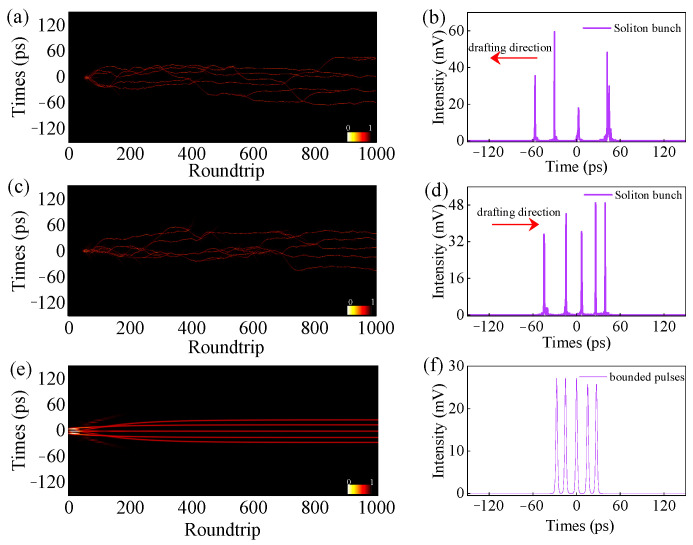
The role of orientation angles in the control of pulse patterns. Pulse pattern evolution and pulse waveform at 1000th roundtrip with (**a**,**b**) (*θ*_1_, *θ*_2_) = (1.03, 0.2), (**c**,**d**) (*θ*_1_, *θ*_2_) = (0.9, 0.1), and (**e**,**f**) (*θ*_1_, *θ*_2_) = (2.31, 1.8), respectively.

Setting Λ as 1.03 × 10^3^ (m·s)^−1^ with different orientation angles *θ*_1_, *θ*_2_, the pulse pattern has diverse bunch states, as shown in Figure 8. Setting (*θ*_1_, *θ*_2_) as (1.03, 0.2), the pulse pattern evolves into a 4-soliton bunch, in which the adjacent pulse temporal intervals gradually decrease at a rate of 6.3 ps at the 1000th roundtrip as shown in Figure 8a,b. By changing the orientation angles, the soliton number increases and the temporal interval changes in reverse direction, as shown in Figure 8c,d. These can be explained by attraction and repulsion forces between solitons [15]. In this condition, shown in Figure 8d, some pulses are phase-locked and have the same interval. When (*θ*_1_, *θ*_2_) are set as (2.31, 1.8), a coherent soliton pattern with five solitons generates, as shown in Figure 8e,f. These pulses are phase-locked, and their temporal interval and intensity are well defined. After several roundtrips, because the pumping exactly compensates for the gain consumed per pulse, they reach a steady state and have the same interval and intensity. With suitable pump power and orientation angles, the pulse number of soliton pattern increases, and the interval of two soliton patterns is the same as that between two adjacent solitons. In other words, harmonic pulses are obtained.

**Figure 9:**
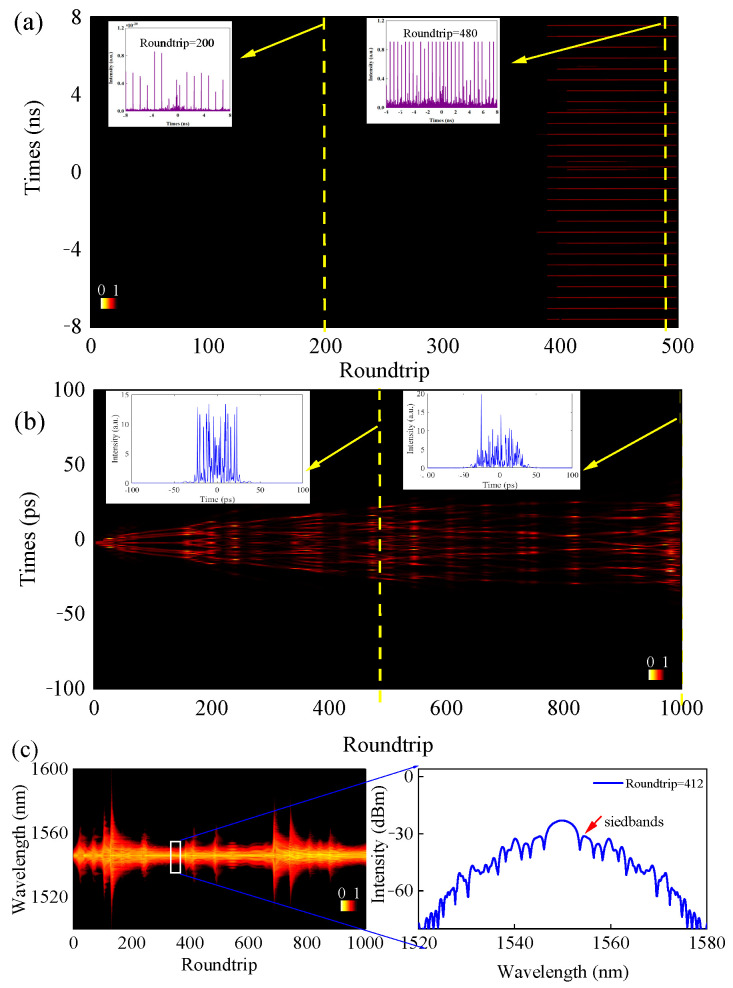
The simulation results. (**a**) 28-pulse bound state. Inset: the temporal coherent pulse pattern on a 16 ns scale at the 200th and 490th roundtrips. (**b**) Dynamics of the h-shaped-like pulse envelope. Inset: the temporal coherent pulse at the 500th and 1000th roundtrips. (**c**) Spectral evolution corresponding to (**b**) and output spectrum at the 362nd roundtrip.

Figure 9a displays an evolution of phase-locked bound pulse pattern with 28 pulses. We can see that almost all pulses have the same intensity and interval. When (*θ*_1_, *θ*_2_) are set as (0.51, 3.12), the evolution of the h-shaped pulse is obtained, as shown in Figure 9b. When the roundtrip is 500, a multi-pulse sequence similar to that shown in Figure 4c is obtained. After the laser pulse resonates in the resonant cavity 1000 times, it outputs a pulse with a h-shaped-like envelope, as shown in the inset of Figure 9b. When the laser output steps into a multi-pulse sequence, the corresponding spectra will display some sidebands, as shown in the enlargement of Figure 9c. The sidebands appear within the 223~389 roundtrips. We also note that the sideband characteristics in the experimental results differ slightly from those in the simulation results. This discrepancy stems from the simulation model not incorporating higher-order nonlinearities and dispersion terms. These simulations display that the two-time scale approach to gain can be used to describe the generation of soliton pattern, soliton bunch, and harmonic waves.

## 4. Discussion

We have experimentally and theoretically investigated the mechanism of the evolution process from h-type pulses to GHz-scale soliton pulses for the first time. Compared with previous reports, the results of this study address the key issue—mechanisms of phase-locked multiple pulses and polarization-dependent gain dynamics. In experiments, h-shaped pulses can evolve into multiple pulses and then transform into phase-locked solitons, as shown in Figure 4. These results confirm the polarization-dependent regulatory properties for arranging the pulses. Previously reported theoretical predictions can only achieve MHz-level repetition frequencies and patterns with a few pulses. In our works, combined with the coupled Ginzburg–Landau equation and the two-time scale approach to gain, the laser physics and the underlying principles of h-shaped pulse and harmonic pulse generation are realized, as shown in Figure 8 and Figure 9. That is to say, these phenomena can be explained by the depletion of gain. The simulation results are matched with the experimental results. This work provides a potential way to gain insight into soliton dynamics in fiber lasers.

## Figures and Tables

**Table 1:** Depletion gain values under different pump and rotation angles.

(*θ*_1_, *θ*_2_)/rad	*N*	Λ*/*(m·s)^−1^	2δG/m^−1^
(2.31, 1.8)	5	1.03 × 10^3^	8.8 × 10^−7^
(0.3, 0.72)	12	1.31 × 10^3^	3.7 × 10^−7^
(1.9, 0.76)	28	7.7 × 10^3^	1.5 × 10^−7^

## Data Availability

The data presented in this study are available on request from the corresponding authors.

## References

[1] Mao D., He Z., Zhang Y., Du Y., Zeng C., Yun L., Luo Z., Li T., Sun Z., Zhao J. (2022). Phase-matching-induced near-chirp-free solitons in normal-dispersion fiber lasers. Light-Sci. Appl..

[2] Yang H.R., Qi M.T., Li X.P., Xue Z., Lu C.H., Cheng J.W., Han D.D., Li L. (2024). Optical nonlinearity of violet phosphorus and applications in fiber laser. Chin. Phys. Lett..

[3] Zeng C., Yang G., Li D., Luo P., Wang R., Du Y., Mao D., Zhao J. (2024). Self-synchronized multi-color Q-switched fiber laser using a parallel-integrated fiber Bragg grating. Chin. Opt. Lett..

[4] Mao D., Wang H., Zhang H., Zeng C., Du Y., He Z., Sun Z., Zhao J. (2021). Synchronized multi-wavelength soliton fiber laser via intracavity group delay modulation. Nat. Commun..

[5] Han Y., Gao B., Wen H., Ma C., Huo J., Li Y., Zhou L., Li Q., Wu G., Liu L. (2024). Pure-high-even-order dispersion bound solitons complexes in ultra-fast fiber lasers. Light-Sci. Appl..

[6] Yang H., Qi M., Li X., Xue Z., Cheng J., Lu C., Han D., Li L., Zhang Y., Zhao F. (2023). Soliton interaction in a MXene-based mode-locked fiber laser. Opt. Express.

[7] Sulimany K., Lib O., Masri G., Klein A., Fridman M., Grelu P., Gat O., Steinberg H. (2018). Bidirectional soliton rain dynamics induced by casimir-like interactions in a graphene mode-locked fiber laser. Phys. Rev. Lett..

[8] Jang J.K., Erkintalo M., Murdoch S.G., Coen S. (2013). Ultraweak long-range interactions of solitons observed over astronomical distances. Nat. Photon..

[9] Chen G.W., Jia K.L., Ji S.K., Zhu J., Li H.Y. (2023). Soliton rains with isolated solitons induced by acoustic waves in a nonlinear multimodal interference-based fiber laser. Laser Phys..

[10] Herink G., Kurtz F., Jalali B., Solli D.R., Ropers C. (2017). Real-time spectral interferometry probes the internal dynamics of femtosecond soliton molecules. Science.

[11] Andrianov A., Kim A. (2021). Widely stretchable soliton crystals in a passively mode-locked fiber laser. Opt. Express.

[12] Komarov A., Leblond H., Sanchez F. (2006). Passive harmonic mode-locking in a fiber laser with nonlinear polarization rotation. Opt. Commun..

[13] Yu C.X., Haus H.A., Ippen E.P., Wong W.S., Sysoliatin A. (2000). Gigahertz-repetition-rate mode-locked fiber laser for continuum generation. Opt. Lett..

[14] Li L., Xue Z., Pang L., Xiao X., Yang H., Zhang J., Zhang Y., Zhao Q., Liu W. (2024). Saturable absorption properties and ultrafast photonics applications of HfS3. Opt. Lett..

[15] Zhao Y., Qin Y., Jia K., Chen L., Chen G., Hu G., Wu T., Li H., He J., Zhou Z. (2024). Dual-comb soliton rains based on polarization multiplexing in a single-walled carbon nanotube mode-locked Er-doped fiber laser. Chin. Opt. Lett..

[16] Zhao J., Li L., Zhao L., Tang D., Shen D. (2018). Cavity-birefringence-dependent h-shaped pulse generation in a thulium-holmium-doped fiber laser. Opt. Lett..

[17] Zheng S., Wang J., Du G., Jiang Y., Yan P., Wang J., Dong F., Lue Q., Guo C., Ruan S. (2023). Frequency domain resolved investigation of unitary h-shaped pulses. Opt. Express.

[18] Yi H., Li X., Zhang J., Zhang X., Ma G. (2024). Higher-order nonlinear effects on optical soliton propagation and their interactions. Chin. Phys. Lett..

[19] Grelu P., Belhache F., Gutty F., Soto-Crespo J.M. (2003). Relative phase locking of pulses in a passively mode-locked fiber laser. J. Opt. Soc. Am. B.

[20] Svelto O., Hanna D.C. (2010). Principles of Lasers.

